# Does Thermoforming Setup Affect Thickness in Thermoplastic Orthodontic Appliances? A Comparison of Single vs. Dual-Model Fabrication

**DOI:** 10.3390/polym18151909

**Published:** 2026-08-04

**Authors:** Ferdi Allaf, Meriç Arslan, Mustafa Özcan, Buket Erdem

**Affiliations:** Department of Orthodontics, Faculty of Dentistry, Istanbul Health and Technology University, Istanbul 34445, Türkiye; ferdi.allaf@istun.edu.tr (F.A.); mustafa.ozcan@istun.edu.tr (M.Ö.); buket.erdem@istun.edu.tr (B.E.)

**Keywords:** clear aligner, thermoforming, thermoplastic, aligner thickness, PET-G

## Abstract

Thermoforming remains the predominant fabrication route for clear thermoplastic orthodontic appliances, yet it reduces and redistributes sheet thickness, which governs force delivered to teeth. Simultaneous thermoforming of two models may reduce fabrication time and material waste; however, its effect on final appliance thickness has not been evaluated. This in vitro study compared appliance thickness after thermoforming over a single model or two models simultaneously, using ten commercial aligner brands with different polymer composition and initial thickness. Thickness was measured with a digital caliper at anterior, canine and posterior sites, and analyzed using three-way ANOVA with Tukey post hoc tests. A significant three-way interaction (measurement point × number of models × brand; *p* < 0.001) was found. Significant single- versus dual-model differences emerged in some brand–region combinations; in most, dual-model appliances were thicker than single-model appliances. Four brands showed no significant difference at any site. Within the limits of this study, simultaneous fabrication of two appliances in a single thermoforming cycle produced appliances with thicknesses comparable to, and often slightly greater than, those fabricated over a single model. Simultaneous thermoforming maintained comparable appliance thickness and may improve manufacturing efficiency.

## 1. Introduction

As patients seeking orthodontic treatment increasingly demand esthetics and comfort, and due to advantages such as shorter chair-side appointments and fewer emergency appointments, the use of clear thermoplastic appliances has become increasingly widespread as an alternative to conventional fixed appliances [1,2].

The standard clear aligner manufacturing process involves acquisition of digital images by intraoral scanning, followed by virtual treatment planning and three-dimensional (3D) printing of a series of orthodontic models. Conventional aligners are then fabricated by thermoforming using either vacuum forming, in which heated biocompatible thermoplastic sheets are adapted to the model under negative pressure, or pressure forming, which uses both positive and negative air pressure to achieve more precise adaptation and contours [3]. However, thermoforming-based aligner fabrication involves a bulky, time-consuming, and costly workflow and contributes substantially to plastic waste generation, for which comprehensive data remain limited [4,5].

Understanding the mechanical behavior of clear appliances, which can be produced from many different thermoplastic materials, is important. These mechanical properties depend on material type, amount of activation and thickness [6]. Variations in appliance thickness can also affect the efficiency of tooth movement [7]. Appliances produced from thinner thermoplastic materials have been reported to generate lower orthodontic forces [6]. The magnitude of the forces can influence biological outcomes such as bone remodeling, cellular damage, hyalinization of the periodontal ligament, bone necrosis and root resorption [8].

It is known that the thermoforming process alters properties of the material such as transparency, rigidity and thickness [9]. Studies have shown that appliance thickness decreases after thermoforming and that this reduction is more pronounced in anterior segments than in posterior segments [10]. Increases in aligner thickness and Young’s modulus have been found to significantly affect the force and moment generated by the appliance, favouring translational tooth movements over tipping movements [11].

According to the literature and manufacturer data, aligner thicknesses generally range between 0.40 mm and 1.50 mm [6,12,13]. For the same material, the transmitted orthodontic force increases as thickness increases [6,12]. Seo et al. reported that thicknesses of 0.5 mm and 0.75 mm are sufficient to generate orthodontic force in the periodontal ligament [14]. A finite element study indicated that 0.75–0.85 mm appliances represent the ideal thickness range for anterior retraction, and that 0.95 mm appliances may be used in situations requiring higher orthodontic forces; however, this use should be regarded only as an exception suitable for a limited number of aligner stages [15].

A stable aligner thickness is important for the accurate application of the forces required for precise tooth movement. Appropriate thickness also improves retention and patient comfort [15]. It additionally affects the durability of the appliance and its resistance to deformation, ensuring uninterrupted continuation of treatment. Thickness control also helps to identify manufacturing errors and to ensure material consistency between different clear appliances. Several methods are used to evaluate aligner thickness, including high-resolution micro-computed tomography (micro-CT), which provides detailed two- and three-dimensional analysis of internal structures and thickness variations; measuring devices for physical measurements; and optical scanners that allow comparison of digital models with CAD designs [8]. The literature has examined the effect of different appliance materials, thicknesses and manufacturing techniques on the physical and mechanical properties of thermoplastic materials [9]. However, most studies have considered only the initial sheet thickness used in production and have not focused on possible thickness changes related to the thermoforming process [16,17,18].

When evaluated in terms of appliance materials, different materials have different molecular properties, which may affect clinical performance [13]. Important advances in polymer science have also influenced the development of aligner materials. Current material formulations aim to improve mechanical, optical and elastomeric properties [19]. Frequently used thermoplastic polymers include polyurethane (PU), polyesters, polyethylene terephthalate (PET) and its glycol-modified derivative PET-G, polycarbonate (PC), thermoplastic polyurethane (TPU) and polypropylene [20]. However, concerns have been raised that these materials may cause environmental and cellular damage through the release of nanoplastics [21].

The clear aligner manufacturing process creates an environmental burden in terms of thermoplastic waste and energy use [22]. For this reason, awareness of the amount of waste resulting from failed prints and post-processing operations, as well as the identification of micro- and nanoplastics that may form during aligner production and use, are regarded as important research areas [21].

In clinical and laboratory practice, simultaneous thermoforming of more than one appliance has been proposed as a practical approach to improve laboratory workflow by enabling the fabrication of two appliances during a single thermoforming cycle, thereby potentially reducing production time. In addition, this approach may reduce manufacturing-related polymer waste by making more efficient use of thermoplastic aligners. However, its effect on the final thickness of thermoformed appliances has not been thoroughly investigated. Therefore, the aim of this study was to comparatively evaluate the effect of thermoforming performed over a single model or over two models on the final aligner thickness across different appliance brands and varying thicknesses.

The null hypothesis was that thermoforming setup (single- or dual-model fabrication), appliance brand, measurement site, and their interactions would not significantly affect the final thickness of thermoformed orthodontic appliances.

## 2. Materials and Methods

**Figure 1:**
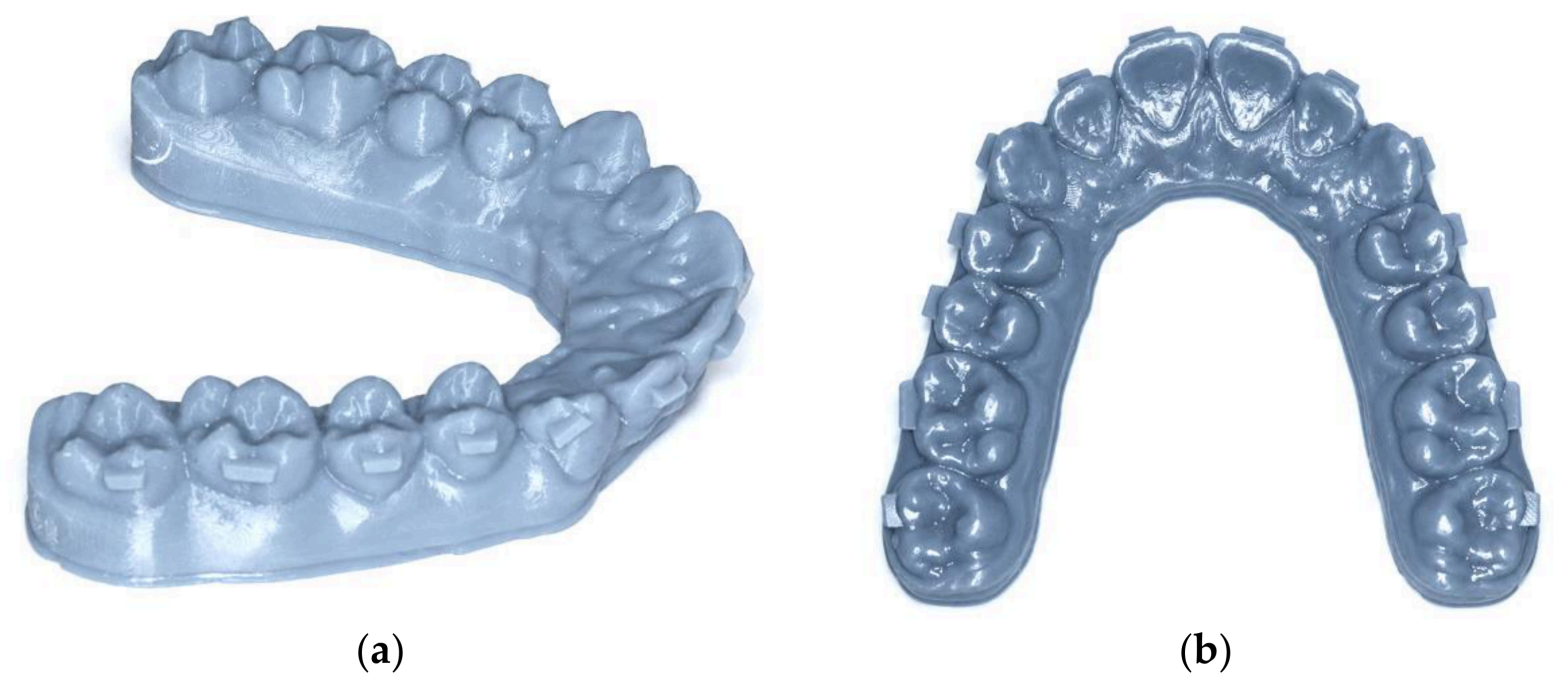
3D-printed resin maxillary model with attachments on the teeth: (**a**) lateral view; (**b**) occlusal view.

In this in vitro study, a 3D-printed resin maxillary model with attachments on the teeth was used (Figure 1). Thermoforming was carried out using twenty identical resin models and ten commercially available aligner materials with different compositions and physical properties. Four commercially available thermoplastic aligner materials were evaluated: MaxFlex (MaxFlex Medical Technology Co., Ltd., Chiayi, Taiwan), Taglus (K Line Industries, Bengaluru, India), Tristar (Whitesmile Clear Pty Ltd., Melbourne, Australia), and Zendura FLX (Bay Materials LLC, Fremont, CA, USA). The commercial thermoplastic orthodontic appliance brands, materials and sheet thicknesses with different properties used in the study are given in Table 1 [23,24,25,26].

The thermoplastic materials were selected to represent a wide range of commercially available aligner materials with different polymer compositions, layer structures, and initial sheet thicknesses, thereby allowing evaluation of the effect of thermoforming setup across materials with diverse physical characteristics.

**Figure 2:**
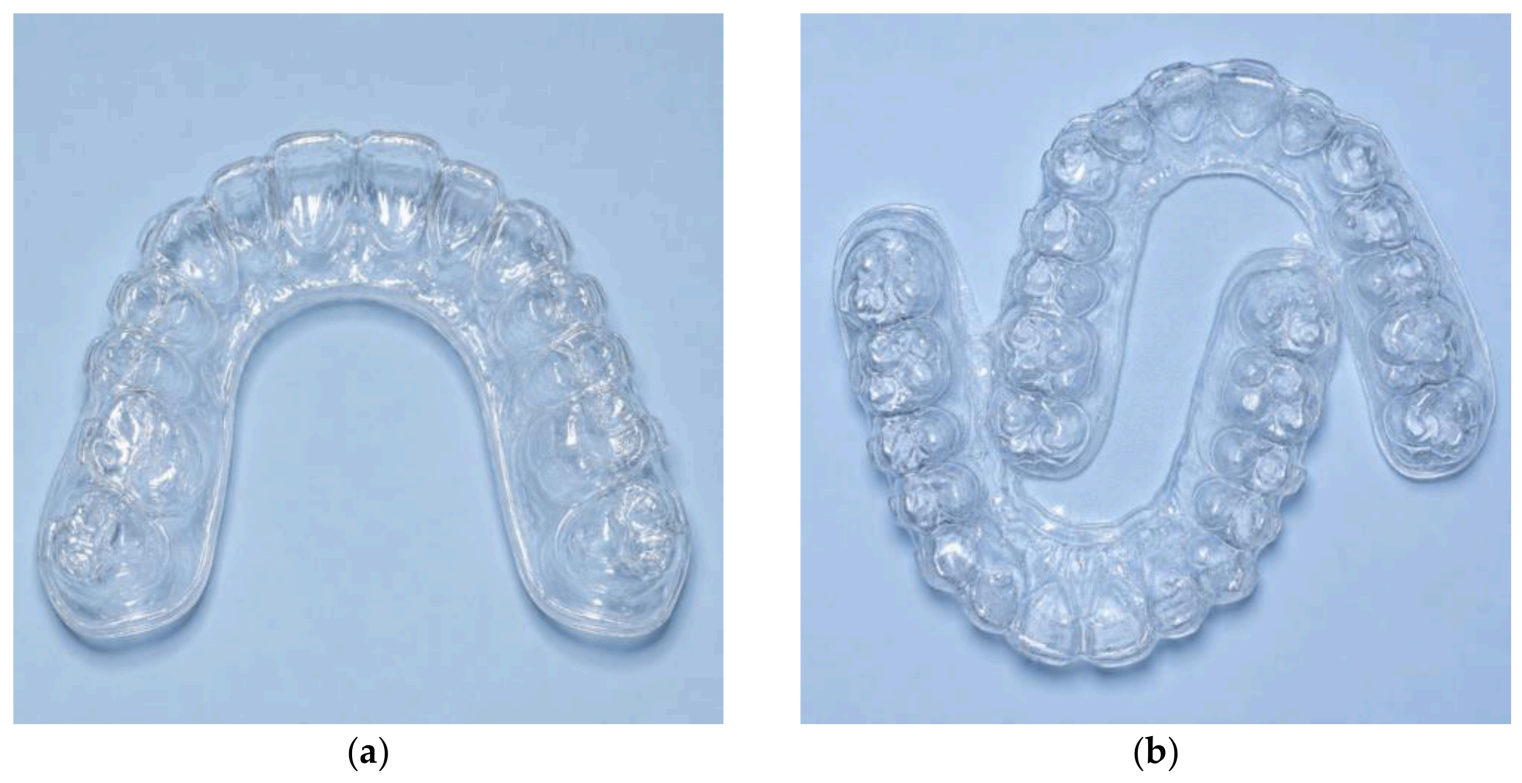
Clear appliances fabricated with the single-model (**a**) and dual-model (**b**) setups.

Thermoforming was carried out on these sheets using two different fabrication protocols: thermoforming over a single model and thermoforming over two models simultaneously. In the single-model group, only one model was placed on the thermoforming device, whereas in the dual-model group two identical maxillary models were positioned symmetrically at the same time. In the dual-model group, the models were positioned as close as possible, with their lingual surfaces partially interlocking, allowing both models to fit simultaneously within the working area of the thermoforming device. They were then positioned centrally within the thermoforming platform, as illustrated in Figure 2b. This configuration resulted in a fixed inter-model distance, which was maintained consistently for all specimens throughout the study.

Thermoforming was performed with a Biostar^®^ (Scheu-Dental, Iserlohn, Germany) pressure thermoforming device. In all procedures, heating time, pressure level and cooling procedures were standardized in accordance with the manufacturer’s recommendations. After thermoforming was completed, the appliances were left to cool at room temperature and were then removed from the models and trimmed (Figure 2).

Appliance thickness measurements were performed by one investigator (F.A.) using a digital caliper. Before thermoforming, all aligner materials were removed from their original packaging and assigned numerical codes. The investigator was blinded to the appliance brands throughout the thermoforming and measurement procedures. However, blinding to the fabrication method during thickness measurements was not feasible because it was apparent whether the appliance had been fabricated over a single or dual model.

The measurement sites were selected to represent clinically relevant regions with different anatomical geometries, where variations in appliance thickness are most likely to occur during thermoforming and can be meaningfully evaluated.

In the posterior region, measurements were taken at the following four points:

- Mesiobuccal cusp tip (A)
- Mesiopalatal cusp tip (B)
- Buccal surface of the attachment (C)
- Gingival surface of the attachment (D)

For the anterior teeth and canines, three reference points were used:

- Midpoint of the incisal edge/cusp tip (A)
- Buccal surface of the attachment (C)
- Gingival surface of the attachment (D)

The measured tooth regions were also classified as the anterior region (incisors), the canine region, and the posterior region (premolars and molars):

- Posterior (X)
- Canine (Y)
- Anterior (Z)

### Statistical Analysis

Each measurement was repeated twice, and the mean of the measurements was used in the statistical analyses. A three-way analysis of variance (3-way ANOVA) was applied to the measurement values to examine the main effects and the interactions of the factors of measurement point/region, model placement type (single vs. dual) and brand. Before analysis, the assumptions of normality and homogeneity of variances were evaluated. The Levene test indicated that variances were not homogeneous between groups (*p* < 0.001). However, because the experimental design was fully balanced (a balanced design with an equal number of observations in each subgroup; error df = 800), the analysis of variance was considered robust to such assumption violations, and Type III ANOVA results were used for the main analyses without any correction. Partial eta squared (ηp^2^) values were calculated to assess effect sizes. To identify the source of significant interactions, subgroup comparisons were performed on Tukey-corrected estimated marginal means (post hoc). Analyses were performed using the R programming language (https://www.r-project.org, R Core Team, Vienna, Austria), and the level of statistical significance was set at *p* < 0.05.

To assess intra-observer reliability, all measurements for five randomly selected appliance brands were repeated by the same investigator two weeks later. The intraclass correlation coefficient (ICC) was calculated to evaluate the agreement between measurements.

## 3. Results

**Figure 3:**
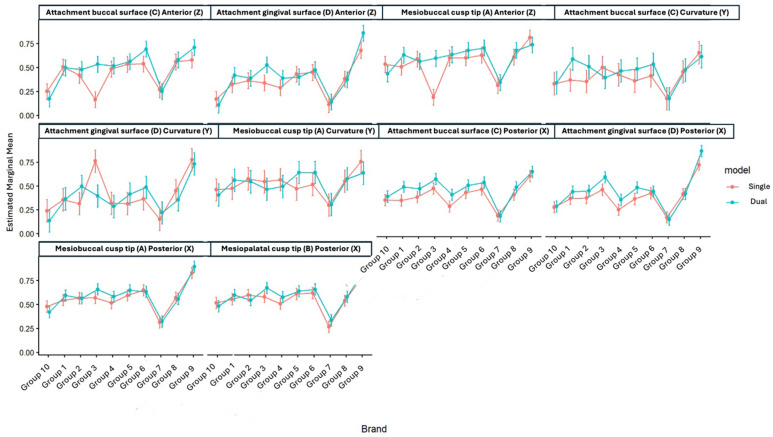
Comparison plot of all data (estimated marginal means for single and dual models across brands and measurement sites).

The analysis showed that the measurement point/region (*p* < 0.001), the number of fabricated models (*p* < 0.001) and the brand used (*p* < 0.001) had statistically significant main effects on the measurement results. The complete dataset is illustrated in Figure 3.

When the combined effects of the independent variables on the measurements were examined, the simultaneous interaction of the three factors was found to be statistically significant at the *p* < 0.001 level (Table 2). Because the presence of a significant three-way interaction makes interpretation of the main effects in isolation statistically misleading, the main effects of number of models, brand and measurement region were not interpreted in general terms.

Instead, in order to examine in detail, the specific conditions under which the difference between appliances fabricated over single and dual models arose, the analysis focused on direct simple-effect (subgroup) analyses and Tukey-corrected post hoc comparisons, shown in Table 3.

When the statistically significant differences were examined, the general trend was that measurements obtained from appliances fabricated over the dual model reached higher values than those over the single model. In particular:

- In Group 3, in the anterior region, very marked differences in favour of the dual model were recorded at the buccal surface of the attachment, the gingival surface of the attachment and the midpoint of the incisal edge.
- In Group 1 and Group 9, significantly higher measurement results were recorded for the dual model in the posterior and anterior region measurements.

However, in Group 3, at the gingival surface of the attachment in the canine region, and contrary to most regions, the values for the single model were found to be statistically higher than those for the dual model.

Intra-observer agreement analysis revealed a high level of agreement between measurements repeated at a two-week interval (Table 4). The mean intra-observer ICC was 0.926 (0.889–0.973).

## 4. Discussion

Although direct 3D aligner fabrication methods are currently developing in clear appliance production, the thermoforming method is still used both in the manufacturing systems of many companies and in in-house aligner production [1]. However, the thermoforming process can lead to a reduction in the initial thermoplastic sheet thickness [27,28]. Golkhani et al. reported that thermoforming reduces material thickness and mechanical strength and, by altering aligner geometry, affects force and torque transmission [29]. Kohda et al. stated that appliances produced from thicker materials generate significantly higher forces than those produced from thinner materials [6]. Seo et al. reported that similar tooth movement and a similar basic stress distribution in the periodontal ligament were obtained with aligners of different thicknesses, although thicker aligners generated only slightly higher loads [14]. By contrast, Kwon et al. showed that thinner materials can transmit higher energy than thicker materials of the same brand [13]. These conflicting findings indicate that the thickness–force relationship varies according to material type and test methodology, further increasing the clinical importance of maintaining thickness in a controlled and predictable manner.

No academic study directly examining the effect of thermoforming over more than one model simultaneously on aligner thickness was found in the literature. Nevertheless, industrial patent applications have emphasized that this method may adversely affect product quality; it has been stated that when production is performed by placing more than one model on a single large thermoforming head, the heated thermoplastic disc is stretched incorrectly, and the final product quality may therefore be inadequate [30]. In light of this information, the present study examined in vitro the effect of single- and dual-model thermoforming conditions on aligner thickness using ten commercial aligner brands with different polymer structures and initial thicknesses. The rationale for investigating simultaneous thermoforming was based on its potential practical application in orthodontic laboratories, where two aligners can be fabricated during a single thermoforming cycle. This approach may improve laboratory workflow and reduce production time per aligner. However, the present study was designed exclusively to evaluate the effect of simultaneous thermoforming on aligner thickness, and no quantitative assessment of fabrication time, material consumption, or cost-effectiveness was performed. Therefore, any potential manufacturing efficiency associated with this approach should be interpreted with caution and requires confirmation in future studies specifically designed to evaluate production efficiency.

Homogeneity of aligner thickness is clinically important; otherwise, the efficiency of some orthodontic tooth movements may decrease. Mantovani et al. examined the thickness homogeneity of thermoformed aligners and showed that thickness was not homogeneous, particularly in the molar regions. They also reported that the lingual-gingival margin thickness was significantly thinner than the occlusal surface [31]. Golkhani et al. examined the effect of the thermoforming process on aligner thickness and showed that thinning of the thermoplastic sheet occurred more on the buccal and lingual surfaces of the teeth than on the occlusal surfaces [29].

In our study, significant thickness differences were observed in the anterior region in Groups 1, 3, 6 and 9; in the posterior region in Groups 1, 3, 4, 5 and 9; and in the canine region in Groups 1 and 3. Among the data with statistically significant differences, the highest thickness difference was detected at the incisal edge in the anterior region in Group 3, reaching approximately 0.40 mm. Previous studies have demonstrated that even relatively small differences in aligner thickness (approximately 0.20–0.25 mm) can significantly influence orthodontic force delivery [18,32]. Therefore, the maximum regional thickness difference of approximately 0.40 mm observed in the present study should not be considered biomechanically negligible and may have meaningful biomechanical implications. However, because force transmission, stress distribution, and tooth movement were not directly evaluated in this study, the biological and clinical significance of this thickness difference cannot be conclusively established based solely on the present findings.

On the other hand, Group 2, Group 7, Group 8 and Group 10 showed no statistically significant difference in any region. In particular, although Group 7 had the thinnest initial sheet (0.30 mm), this finding should not be interpreted as evidence that very thin materials are inherently insensitive to changes in thermoforming geometry. Since the polymer composition of this material was not disclosed by the manufacturer and only one material with this thickness was evaluated, the observed behavior may reflect material-specific characteristics rather than the effect of thickness alone. Further studies using identical materials with different initial thicknesses are required to clarify the independent effect of material thickness.

Additionally, the stable findings observed in Group 8 are thought to be related to the hybrid multilayer structure of the material. Krey et al. reported that multilayer materials exhibit a more homogeneous thickness distribution during the thermoforming process than single-layer materials [33]. The attachment surfaces were the most frequently affected location in all three regions, whereas differences at the cusp tip or incisal edge were limited only to the anterior region.

In the findings obtained in this study, the thickness values measured in appliances fabricated under the dual-model condition were found to be higher than those under the single-model condition. This finding suggests that, when a single thermoplastic sheet is stretched simultaneously over two models, the geometry of the plastic deformation changes and the material is subjected to less elongation over each individual model. This is similar to the findings of Ghoraba et al., who reported that aligner thickness progressively decreased as model height increased [34]. The only exception was observed in Group 3, at the gingival surface of the attachment on the canine tooth in the canine region, where appliance thickness was greater for the single model. This suggests that the rigid structure of PET-G together with its high initial thickness (1.02 mm) may have restricted geometric flow, predisposing to accumulation of thickness at this specific location in the single model.

Nevertheless, the increase in thickness observed in our study did not occur to a similar degree in all brand and region combinations. The most marked differences were observed in PET-G-based Group 3. Krey et al. reported that PET and PU materials experience thickness loss in a similar pattern during thermoforming; however, thinning reaching approximately 50% of the initial thickness was observed in the buccal and gingival regions. Emphasizing that halving the layer thickness can reduce the moment of resistance to one quarter, the researchers stated that this situation may particularly complicate the application of torque movements [33]. Staderini et al. showed that, after thermoforming, thickness decreased by 15% and weight by 11% in PET-G material; however, no significant change was observed in elastic modulus or tensile strength [35]. These findings suggest that the response of PET-G material to the thermoforming process differs from that of TPU and that the mechanical consequences of thickness loss vary according to material type.

In the brand-based evaluation, the most marked differences were observed in Group 3, Group 9 and Group 1. In Group 3, the thickness differences measured at the buccal surface of the attachment and at the incisal edge tip in the anterior region reached 0.37 mm and 0.41 mm, respectively, corresponding to approximately one third of the initial thickness. This finding is thought to be explained by the combined effect of the rigid polymer structure of PET-G restricting plastic flow, the high initial thickness increasing sensitivity to changes in thermoforming geometry, and the anterior incisal tooth tip constituting a geometrically critical location in the arch form. Gowardhan et al., on the other hand, showed that TPU material experienced less thickness loss than PET-G; however, unlike the results of our study, they reported that the difference between the two materials was significant in the molar region and disappeared in the anterior region [8].

In terms of regional distribution, thickness differences appeared more frequently and more markedly in the anterior and canine regions than in the posterior region. This pattern is consistent with the literature findings that thermoplastic material is subjected to greater stretching in regions of high curvature during thermoforming. Krey et al. reported that thickness loss showed a continuous increase from incisal to gingival and that this reduction occurred predominantly on the buccal surfaces [33]. Similarly, Ghoraba et al. showed that thickness differences became more marked on the buccal surfaces and remained more limited on the lingual surface and at the incisal tip [34]. In the finite element analysis of Luo et al., the mean thickness thinning in the buccal and lingual regions was shown to be significantly higher than in the occlusal region, and it was numerically confirmed that regions of high curvature are subjected to greater stretching during thermoforming [36]. Gowardhan et al. also confirmed the posterior–anterior thickness gradient with nano-CT, reporting that aligner thickness decreased from posterior to anterior, while adaptation in the anterior region was better [8].

In our study, the appliance thickness at the gingival surface of the attachment was found to be affected in some groups. Taheri et al. showed that PET-G-based material exhibited greater volumetric change at the attachment region than TPU and emphasized that attachment geometry increased local stress concentration [37]. In this study, a statistically significant increase in thickness was detected at the gingival surface of the attachment in the anterior and posterior regions in appliances fabricated under the dual-model condition, particularly in Group 3 and Group 9, which had the highest initial thicknesses. Considering that a reduction in thickness at the gingival surface of the attachment may adversely affect extrusion movements, the preservation or increase in thickness in this region under the dual-model condition may be regarded as a clinically favourable finding.

The limitations of this study should be considered. A single type of resin model was used for standardization, and it should be borne in mind that different arch forms or tooth anatomies could affect the results. Thickness measurements were performed with a digital caliper. Consequently, very small regional thickness variations may not have been detected, and this should be considered when interpreting the findings. Although this method is widely used in clinical practice, it offers more limited precision compared with advanced imaging methods such as micro-CT or nano-CT.

All thermoforming procedures were performed with a single pressure thermoforming device (Biostar^®^). The demonstration by de Moraes et al. that different thermoforming systems significantly affect material properties indicates that caution should be exercised in generalizing the findings to different device conditions [38]. Although the distance between the models in the dual-model group was fixed across all specimens, it should be considered that this standardization may not always be achievable under clinical and laboratory conditions.

Finally, because of the in vitro design of the study, the effects of intraoral factors such as the saliva environment, occlusal forces and thermal cycling could not be evaluated. In addition, force transmission, stress distribution, and actual tooth movement were not directly assessed. Therefore, although the observed regional thickness differences may have biomechanical implications, their biological and clinical significance cannot be conclusively established based solely on the present findings. Further studies integrating thickness measurements with mechanical testing, force analysis, and tooth movement evaluation are needed to determine the clinical relevance of these thickness variations. For this reason, caution should be exercised in directly transferring the findings to clinical conditions.

## 5. Conclusions

For different appliance brands and thicknesses, after thermoforming performed over a single model versus over two side-by-side models, no difference in appliance thickness was found in some groups and regions, whereas a difference was found in others. Therefore, the null hypothesis was partially rejected. In the groups where a difference was found, the thickness values obtained from appliances fabricated over the dual model were generally higher than those obtained from appliances fabricated over the single model.

The findings of the study demonstrate that fabricating two appliances simultaneously during the same thermoforming process yields acceptable results in terms of appliance thickness and support the conclusion that this approach should be regarded as a viable method for clinical and laboratory application.

## Figures and Tables

**Table 1:** Brands, polymer compositions and initial thicknesses of the thermoplastic appliance materials used in the study.

Group	Brand Name	Polymer	Sheet Thickness
Group 1	Maxflex	TPU	0.76 mm
Group 2	Taglus Premium	PET-G	0.80 mm
Group 3	Taglus Premium	PET-G	1.02 mm
Group 4	Taglus Pu Flex	PU	0.76 mm
Group 5	Taglus Standard	Copolyester	0.80 mm
Group 6	Taglus Tuff	Copolyester	0.80 mm
Group 7	Tristar Attachment Template	Not disclosed by the manufacturer	0.30 mm
Group 8	Tristar TruBalance	PET-G + TPU	0.76 mm
Group 9	Tristar T-Plus	TPU	1.80 mm
Group 10	Zendura	TPU + Polyester	0.76 mm

**Table 2:** Three-way ANOVA results for the main effects and interactions on the measurement values.

Comparison	F Value	*p* Value	*ηp* ^2^
Measurement point × Number of models	2.20	0.020	0.02
Measurement point × Brand	4.21	<0.001	0.30
Number of models × Brand	4.05	<0.001	0.04
Measurement point × Model × Brand	1.93	<0.001	0.16

**Table 3:** Statistically significant differences between single- and dual-model fabrication across brands and regions.

Brand (Group)	Region and Point	Model (Single − Dual)	SE	*p* Value
Group 3	Attachment buccal surface (C)—Anterior (Z)	−0.370	0.059	<0.001
Group 6	Attachment buccal surface (C)—Anterior (Z)	−0.155	0.059	0.009
Group 9	Attachment buccal surface (C)—Anterior (Z)	−0.130	0.059	0.029
Group 3	Attachment gingival surface (D)—Anterior (Z)	−0.187	0.059	0.001
Group 9	Attachment gingival surface (D)—Anterior (Z)	−0.180	0.059	0.002
Group 1	Incisal edge midpoint (A)—Anterior (Z)	−0.125	0.059	0.036
Group 3	Incisal edge midpoint (A)—Anterior (Z)	−0.407	0.059	<0.001
Group 1	Attachment buccal surface (C)—Canine (Y)	−0.220	0.084	0.009
Group 3	Attachment gingival surface (D)—Canine (Y)	+0.365	0.084	<0.001
Group 1	Attachment buccal surface (C)—Posterior (X)	−0.141	0.042	<0.001
Group 4	Attachment buccal surface (C)—Posterior (X)	−0.123	0.042	0.003
Group 3	Attachment gingival surface (D)—Posterior (X)	−0.130	0.042	0.002
Group 5	Attachment gingival surface (D)—Posterior (X)	−0.120	0.042	0.004
Group 9	Attachment gingival surface (D)—Posterior (X)	−0.142	0.042	<0.001

Negative values indicate that, at the relevant location, measurements from the dual-model setup were statistically higher than those from the single-model setup. Only significant comparisons with *p* < 0.05 are listed.

**Table 4:** Intra-observer reliability of thickness measurements assessed using the intraclass correlation coefficient (ICC).

Group	ICC
Group 1	0.894
Group 2	0.973
Group 3	0.969
Group 4	0.889
Group 5	0.907

## Data Availability

Data are contained within the article. Further inquiries can be directed to the corresponding author.

## References

[1] Bandić R., Vodanović K., Vuković Kekez I., Medvedec Mikić I., Galić I., Kalibović Govorko D. (2024). Thickness variations of thermoformed and 3D-printed clear aligners. Acta Stomatol. Croat..

[2] Rossini G., Parrini S., Castroflorio T., Deregibus A., Debernardi C.L. (2015). Efficacy of clear aligners in controlling orthodontic tooth movement: A systematic review. Angle Orthod..

[3] Ho C.-T., Huang Y.-T., Chao C.-W., Huang T.-H., Kao C.-T. (2021). Effects of different aligner materials and attachments on orthodontic behavior. J. Dent. Sci..

[4] Macrì M., Murmura G., Varvara G., Traini T., Festa F. (2022). Clinical performances and biological features of clear aligners materials in orthodontics. Front. Mater..

[5] Putrino A., Barbato E., Galluccio G. (2021). Clear aligners: Between evolution and efficiency—A scoping review. Int. J. Environ. Res. Public Health.

[6] Kohda N., Iijima M., Muguruma T., Brantley W.A., Ahluwalia K.S., Mizoguchi I. (2013). Effects of mechanical properties of thermoplastic materials on the initial force of thermoplastic appliances. Angle Orthod..

[7] KrishnaKailash S.D., Roychoudhury S., Amit K., Basu P., Santosh G.K., Yousuf K.N. (2024). Assessment of change in thickness of aligner using vacuum and pressure thermoforming techniques: An in-vitro study. J. Adv. Med. Dent. Sci. Res..

[8] Gowardhan S., Krishnakumaran M., Krishnan B., Arumugam S.P., Sekar A.S., Subashree R. (2025). Measurement of aligner thickness and gap width in two types of clear aligner sheets manufactured using two different thermoforming machines—A nano-CT pilot study. Turk. J. Orthod..

[9] Ryu J.-H., Kwon J.-S., Jiang H.B., Cha J.-Y., Kim K.-M. (2018). Effects of thermoforming on the physical and mechanical properties of thermoplastic materials for transparent orthodontic aligners. Korean J. Orthod..

[10] Walton D.K., Fields H.W., Johnston W.M., Rosenstiel S.F., Firestone A.R., Christensen J.C. (2010). Orthodontic appliance preferences of children and adolescents. Am. J. Orthod. Dentofac. Orthop..

[11] Ahmad W., Xiong J., Xia Z. (2025). The mechanical effect of aligner’s thickness and material properties in invisible orthodontics: A quantitative finite element study. Dent. Mater. J..

[12] Liu D.-S., Chen Y.-T. (2015). Effect of thermoplastic appliance thickness on initial stress distribution in periodontal ligament. Adv. Mech. Eng..

[13] Kwon J.-S., Lee Y.-K., Lim B.-S., Lim Y.-K. (2008). Force delivery properties of thermoplastic orthodontic materials. Am. J. Orthod. Dentofac. Orthop..

[14] Seo J.-H., Eghan-Acquah E., Kim M.-S., Lee J.-H., Jeong Y.-H., Jung T.-G., Hong M., Kim W.-H., Kim B., Lee S.-J. (2021). Comparative analysis of stress in the periodontal ligament and center of rotation in the tooth after orthodontic treatment depending on clear aligner thickness—Finite element analysis study. Materials.

[15] Alhasyimi A.A., Ayub A., Farmasyanti C.A. (2024). Effectiveness of the attachment design and thickness of clear aligners during orthodontic anterior retraction: Finite element analysis. Eur. J. Dent..

[16] Elkholy F., Mikhaiel B., Schmidt F., Lapatki B. (2017). Mechanical load exerted by PET-G aligners during mesial and distal derotation of a mandibular canine: An in vitro study. J. Orofac. Orthop..

[17] Elkholy F., Schmidt F., Jäger R., Lapatki B.G. (2017). Forces and moments applied during derotation of a maxillary central incisor with thinner aligners: An in-vitro study. Am. J. Orthod. Dentofac. Orthop..

[18] Elkholy F., Schmidt F., Jäger R., Lapatki B.G. (2016). Forces and moments delivered by novel, thinner PET-G aligners during labiopalatal bodily movement of a maxillary central incisor: An in vitro study. Angle Orthod..

[19] Bichu Y.M., Alwafi A., Liu X., Andrews J., Ludwig B., Bichu A.Y., Zou B. (2023). Advances in orthodontic clear aligner materials. Bioact. Mater..

[20] Ravuri P., Kubavat A.K., Rathi V., John T.L., Varma P.K., Mujoo S., Somaraj V. (2024). Effectiveness and biocompatibility of tooth aligners made from polyethylene terephthalate glycol (PET-G), polypropylene (PP), polycarbonate (PC), thermoplastic polyurethanes (TPUs), and ethylene-vinyl acetate (EVA): A systematic review. J. Pharm. Bioallied Sci..

[21] Macrì M., D’Albis V., Marciani R., Nardella M., Festa F. (2024). Towards sustainable orthodontics: Environmental implications and strategies for clear aligner therapy. Materials.

[22] Caelli C., Tamburrino F., Brondi C., Razionale A.V., Ballarino A., Barone S. (2023). Sustainability in healthcare sector: The dental aligners case. Sustainability.

[23] Taglus (2026). Taglus Aligner Sheets. https://www.taglus.com/.

[24] Tristar (2026). TRISTAR Clear Aligner Materials. https://alignermaterial.com/.

[25] Shanghai Maxflex Medical Technology Co., Ltd. (2026). Aligner Materials. https://www.maxflexaligner.com/.

[26] Dental Z. (2026). Zendura. https://www.zenduradental.com/?srsltid=AfmBOoqwG-AK2jA1cdbC78C1OWb0UQD_Bz7N9l7Xxe9LoiBDLUmAU704.

[27] Bucci R., Rongo R., Levatè C., Michelotti A., Barone S., Razionale A.V., D’Antò V. (2019). Thickness of orthodontic clear aligners after thermoforming and after 10 days of intraoral exposure: A prospective clinical study. Prog. Orthod..

[28] Lombardo L., Palone M., Longo M., Arveda N., Nacucchi M., De Pascalis F., Spedicato G.A., Siciliani G. (2020). MicroCT X-ray comparison of aligner gap and thickness of six brands of aligners: An in-vitro study. Prog. Orthod..

[29] Golkhani B., Weber A., Keilig L., Reimann S., Bourauel C. (2022). Variation of the modulus of elasticity of aligner foil sheet materials due to thermoforming. J. Orofac. Orthop..

[30] Vitiello C. (2025). Apparatus for Molding Dental Aligners. US Patent.

[31] Mantovani E., Parrini S., Coda E., Cugliari G., Scotti N., Pasqualini D., Deregibus A., Castroflorio T. (2021). Micro computed tomography evaluation of Invisalign aligner thickness homogeneity. Angle Orthod..

[32] Hahn W., Dathe H., Fialka-Fricke J., Fricke-Zech S., Zapf A., Kubein-Meesenburg D., Sadat-Khonsari R. (2009). Influence of thermoplastic appliance thickness on the magnitude of force delivered to a maxillary central incisor during tipping. Am. J. Orthod. Dentofac. Orthop..

[33] Krey K.-F., Behyar M., Hartmann M., Corteville F., Ratzmann A. (2019). Behaviour of monolayer and multilayer foils in the aligner thermoforming process. J. Aligner Orthod..

[34] Ghoraba O., Bourauel C., Aldesoki M., Singer L., Ismail A.M., Elattar H., Alhotan A., Elshazly T.M. (2024). Effect of the height of a 3D-printed model on the force transmission and thickness of thermoformed orthodontic aligners. Materials.

[35] Staderini E., Chiusolo G., Guglielmi F., Papi M., Perini G., Tepedino M., Gallenzi P. (2024). Effects of thermoforming on the mechanical, optical, chemical, and morphological properties of PET-G: In vitro study. Polymers.

[36] Luo L., Zhang H., Li X., Zou Y. (2025). Predicting thickness distribution of thermoplastic dental aligners via KBKZ-based finite element analysis. Sci. Rep..

[37] Taheri R., García-Marín C., Aizpuru-Arotzena M., Iglesias-Linares A. (2026). 3D volumetric changes and force delivery at the attachment region of different aligner materials. Clin. Oral Investig..

[38] de Moraes K.C., Facury A.G.B.F., Rockenbach P.A., Neto E.P.B., da Gloria Bellotti M., Correr-Sobrinho L., Costa A.R. (2026). Device-dependent thermoforming effects on surface roughness and Knoop microhardness of thermoplastic aligner materials: An in vitro study. Int. Orthod..

